# A Case of Sodium Chlorite Toxicity Managed with Concurrent Renal Replacement Therapy and Red Cell Exchange

**DOI:** 10.1007/s13181-012-0256-9

**Published:** 2012-09-21

**Authors:** Adam Romanovsky, Dennis Djogovic, Dat Chin

**Affiliations:** 1 Division of Critical Care Medicine, University of Alberta, Edmonton, AB Canada; 2 Division of Nephrology, University of Alberta, Edmonton, AB Canada; 3 Department of Emergency Medicine, University of Alberta, Edmonton, AB Canada; 4 General Systems Intensive Care Unit, University of Alberta Hospital, 3C1.12 WMC, 8440 112 Street, Edmonton, AB T6G 2B7 Canada

**Keywords:** Sodium chlorite, Renal replacement therapy, Red cell exchange, Plasma exchange, Oxidative hemolysis, Acute kidney injury

## Abstract

**Introduction:**

Sodium chlorite is a powerful oxidizing agent with multiple commercial applications. We report the presentation and management of a single case of human toxicity of sodium chlorite.

**Case report:**

A 65-year-old man presented to hospital after accidentally ingesting a small amount of a sodium chlorite solution. His principal manifestations were mild methemoglobinemia, severe oxidative hemolysis, disseminated intravascular coagulation, and anuric acute kidney injury. He was managed with intermittent hemodialysis, followed by continuous venovenous hemofiltration for management of acute kidney injury and in an effort to remove free plasma chlorite. Concurrently, he underwent two red cell exchanges, as well as a plasma exchange, to reduce the burden of red cells affected by chlorite. These interventions resulted in the cessation of hemolysis with stabilization of serum hemoglobin and platelets. The patient survived and subsequently recovered normal renal function.

**Discussion:**

This is only the second case of sodium chlorite intoxication reported in the medical literature and the first to report the use of renal replacement therapy in combination with red cell exchange in its management.

## Introduction

Sodium chlorite is a powerful oxidizing agent used to generate chlorine dioxide, which has several applications, including bleaching of pulp, paper, and textiles. When ingested, oxidizing agents can lead to methemoglobinemia, and intravascular hemolysis may ensue. Only one case of sodium chlorite ingestion has been reported in the literature [1]. We report the only known case of accidental sodium chlorite ingestion leading to mild methemoglobinemia, severe hemolysis, and acute kidney injury (AKI) successfully treated with hemodialysis, followed by hemofiltration and concurrent red cell and plasma exchange.

## Case

A 65-year-old man presented to his local hospital with nausea, vomiting, diarrhea, and dark urine after ingesting a small amount of a 28 % sodium chlorite solution. He had diluted the sodium chlorite in a cup with an unmeasured amount of water and was using this solution to clean his fruit. He then accidentally drank a mouthful of the solution after confusing this cup with another that contained only water. Upon ingestion, he immediately self-induced vomiting, and only after 4 h when he noticed dusky finger tips and lips, did he present to the hospital. He was transferred to our institution and, upon arrival, was hemodynamically stable, mildly confused, and anuric. Initial pertinent laboratory results revealed hemoglobin 184 g/L, white blood cell count 22.1 × 10^9^/L, normal platelet count, creatinine 144 μmol/L, sodium 141 mmol/L, potassium 5.5 mmol/L, bicarbonate 19 mmol/L, chloride 114 mmol/L, calculated anion gap 14, ionized calcium 1.13 mmol/L, phosphate 1.37 mmol/L, and haptoglobin of <0.08 g/L. Bilirubin and lactate dehydrogenase were unable to be processed by the laboratory, but the serum was described as being brownish in color. Urine was brown, and examination showed 3^+^ hemoglobin, with few red blood cells and many hemegranular casts. Initial arterial blood gas showed pH 7.35, p_a_CO_2_ 35 mmHg, p_a_O_2_ 256 mmHg, methemoglobin (MetHb) 6.7 %, and lactate 1.8 mmol/L.

He was admitted to the intensive care unit and treated with an 8-h session of hemodialysis to manage hyperkalemia and to attempt removal of sodium chlorite. We used a high-flux dialysis filter (Toray TS-1.6SL polysulfone filter) and prescribed a blood flow of 400 mL/min with a dialysate flow of 500 mL/min to maximize clearance. The dialysate contained electrolytes in the following concentrations: bicarbonate 35 mmol/L, sodium 140 mmol/L, potassium 2 mmol/L, magnesium 0.75 mmol/L, calcium 1.5 mmol/L, and glucose 8 mmol/L.

He was not treated with methylene blue or ascorbic acid but was started on a high-dose *N*-acetylcysteine infusion. We used the 21-h intravenous *N*-acetylcysteine regimen recommended in acetaminophen toxicity (150 mg/kg over 1 h, followed by 50 mg/kg over 4 h then 100 mg/kg over 16 h). While on dialysis, his MetHb levels decreased to normal, but upon its completion, he developed an increasing lactate (8.2 mmol/L), a drop in hemoglobin to 87 g/L, and hemodynamic instability requiring intravenous fluid resuscitation and vasopressor support with norepinephrine (10 mcg/min). A serum glucose-6-phosphate dehydrogenase (G6PD) screen revealed deficiency, and plasma-free hemoglobin was markedly elevated at 1,783 mg/L. A peripheral blood film showed blister and bite cells consistent with oxidative hemolysis, with no evidence of microangiopathic hemolysis. It also showed marked thrombocytopenia due to peripheral consumption with platelets of 28 × 10^6^/L. In addition to thrombocytopenia, elevated PT INR (1.4) and D-dimer (15.43 mg/L) were consistent with the diagnosis of disseminated intravascular coagulation (DIC). Peak fibrinogen and creatine kinase were 2.2 g/L and 2,926 U/L, respectively.

**Fig. 1:**
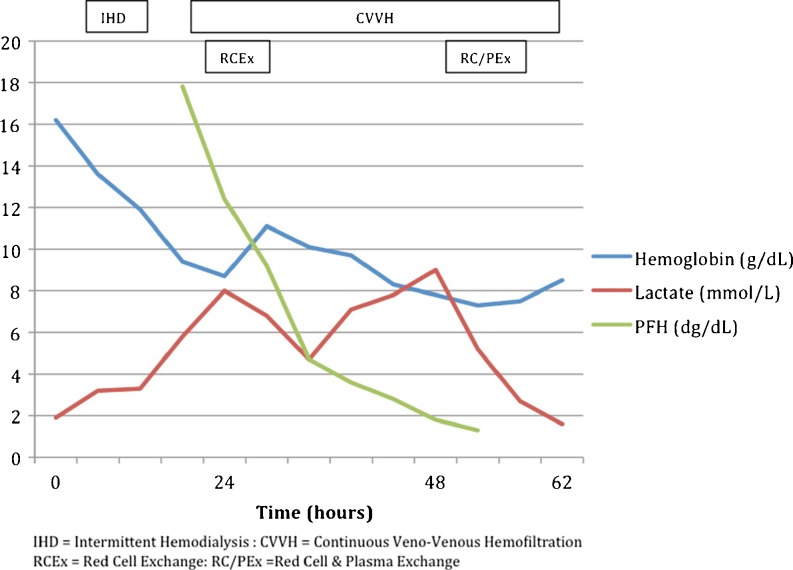
Serum hemoglobin, lactate, and plasma-free hemoglobin (PFH) over time

High FiO_2_ was delivered via a non-rebreather mask, and continuous venovenous hemofiltration (CVVH) with a total effluent of 50 mL/kg was initiated. The filter used was Gambro ST100 AN69® membrane, and replacement fluid was Gambro PrismaSol® with electrolyte concentrations as follows: bicarbonate 32 mmol/L, sodium 140 mmol/L, potassium 3 mmol/L, chloride 108 mmol/L, magnesium 0.5 mmol/L, and lactate 3.0 mmol/L. Concurrently, red cell exchange was initiated via a separate 12 French dual lumen central venous catheter. Upon completion of the 2-h long red cell exchange (total of 3.3 L exchanged), his symptoms improved, and the lactate and plasma-free hemoglobin decreased to 4.8 mmol/L and 967 mg/L, respectively, (Fig. 1). After an initial several hours of stability, his hemoglobin again dropped to 83 g/L. This was accompanied by worsening nausea and back pain, as well as an increase in lactate to 9.5 mmol/L and a recurrence of hemodynamic instability. He underwent a second session of red cell exchange (2 h with total exchange of 2 L), which was followed by plasma exchange (3.5 h with 5.2 L exchanged). With this exchange, his symptoms again abated, and his lactate normalized. Plasma-free hemoglobin decreased to 125 mg/L.

He subsequently remained stable with no further drops in hemoglobin or rises in lactate. He underwent esophagogastroduodenoscopy which revealed only superficial gastric ulcers. Repeat screens for G6PD deficiency at 4 days and 12 months were negative. He was continued on continuous renal replacement for 96 h in total at which time he was transitioned to intermittent hemodialysis as he remained anuric; 17 days after admission to hospital, he was no longer dialysis-dependent and was discharged home. Over the following 2 months, his serum creatinine returned to its premorbid level of 94 μmol/L.

## Discussion

Sodium chlorite is a white crystal that is readily dissolvable in water. Its primary commercial application is that of a bleaching agent in the pulp and paper and textile industries. Outside of its commercial uses, it has numerous unfounded claims as a “natural health” cure for a multitude of health issues.

Although there is not an abundance of data regarding the physiologic effects of sodium chlorite, several animal and human studies have documented its ability to cause methemoglobinemia, hemolysis, and glutathione depletion [2]. More physiologic data exist for the related compound, sodium chlorate [3, 4]. A study by Singelmann et al. examined the effect of incubating human red cells with varying concentrations of chlorate, showing that these red cells develop glutathione depletion and methemoglobinemia. In addition, chlorate led to inhibition of G6PD and increased membrane rigidity. This drug-induced G6PD deficiency, in addition to glutathione depletion and membrane rigidity, leads to oxidative hemolysis and explains the lack of efficacy of methylene blue in these patients [3, 4]. Importantly, Steffen et al. noted that the presence of methemoglobin was necessary for inhibition of G6PD and cross-linking of membrane proteins. If, however, hypochlorite was used instead of chlorate; oxidation of hemoglobin and oxidative hemolysis occurred independent of methemoglobin. This feature may explain the severity of hemolysis in our case, despite the presence of only mild methemoglobinemia.

Only one previous case of acute sodium chlorite toxicity has been reported in the literature [1]. Lin et al. described a case of intentional ingestion of sodium chlorite leading to severe methemoglobinemia, hemolysis, DIC, and renal failure. The patient presented with a MetHb level of 59 %, which did not respond to administration of multiple doses of methylene blue. He subsequently developed hemolysis and DIC and was treated with continuous arteriovenous hemofiltration. He survived and eventually recovered renal function.

Our patient presented with similar gastric upset, likely as a result of chlorite-induced irritation of the gastric mucosa. Unlike the previous case, our patient presented with only mild methemoglobinemia. Despite this, he developed inhibition of G6PD and went on to suffer life-threatening oxidative hemolysis. This suggests that methemoglobinemia is not a prerequisite for chlorite-induced enzyme inhibition and membrane protein cross-linking. Due to the low levels of MetHb, acute G6PD deficiency, and previous lack of efficacy reported for chlorate and chlorite ingestions, we chose not to administer methylene blue or ascorbic acid. We did, however, employ high-dose *N*-acetylcysteine in an attempt to replenish depleted glutathione stores. Although we had no data to suggest its use, we felt that there was a theoretical benefit with minimal risk of adverse consequences. It is unclear what factors contributed to the discrepancy between MetHb levels and hemolysis in the two cases. It may be that a smaller ingested amount of chlorite resulted in less MetHb, although this leaves unexplained the similar degree of hemolysis in the two cases. It is plausible, as explained above, that oxidative hemolysis with chlorite may occur independently of methemoglobinemia.

In the absence of severe methemoglobinemia, we believe that the intermittent lactic acidosis resulted from a combination of decreased oxygen-carrying capacity and reduced tissue perfusion. The patient had hemolyzed more than half of his red cells, thereby reducing oxygen-carrying capacity of more than 50 %. In addition, massive hemolysis can lead to hypotension due to peripheral vasodilation, thereby leading to reduced tissue perfusion and lactic acidosis [5]. This explains the simultaneous occurrence of hemolysis, hypotension, and increasing serum lactate levels.

The exact cause of the acute kidney injury is unclear but is likely multifactorial. Lin et al. reported a diagnosis of acute interstitial nephritis in a patient with sodium chlorite toxicity [1]. Findings on renal biopsy included acute tubulointerstitial changes, lymphocytic infiltration, and severe interstitial edema. They also described moderate tubule damage with hemorrhage and necrosis along with focally collapsed loops. However, several of these findings, such as necrosis, are not usual findings of interstitial nephritis. In addition, the extremely rapid progression to anuria is not consistent with interstitial nephritis.

Several studies of chlorate, a chemically similar compound to chlorite, suggest that renal toxicity is due to a combination of methemoglobinuria, direct proximal tubular toxicity, and possibly lesions similar to those seen with the hemolytic uremic syndrome [4, 6]. In the absence of severe methemoglobinemia, it is likely that the patient’s AKI is accounted for by a combination of tubular toxicity from hemoglobin and renal ischemia due to poor oxygen delivery. The renal medulla has a very low oxygen tension in normal conditions and is, therefore, extremely sensitive to reduced systemic oxygen delivery. Cases of renal failure with other forms of oxidative hemolysis support this theory [7].

With a molecular weight of 67.45 daltons, unbound chlorite should be effectively removed by hemodialysis with a standard or high-flux dialysis membrane. Our patient’s hemoglobin and hemodynamics remained stable while receiving IHD, which we theorize may be related to removal of free chlorite. It is important to note that the amount of free versus bound chlorite in serum is unknown, and that hemodialysis was undertaken both to manage AKI and in an attempt to remove any free chlorite. It is difficult to know whether or not extracorporeal removal of chlorite played a significant role in improving the patient’s outcome, and it remains possible that it was of little benefit.

In trying to remove small toxins, such as chlorite, high efficiency clearance achieved with intermittent hemodialysis dictates that this be the modality of choice. The volume of distribution of chlorite is unknown; therefore, a prolonged session of intermittent dialysis, similar to that used in a lithium overdose, is prudent.

Our patient developed worsening hemolysis after completion of a session of intermittent hemodialysis, which we suspected was due to a rebound increase in serum chlorite levels secondary to redistribution from the extravascular space. Due to intermittent hemodynamic instability and in an attempt to avoid rebound increases in serum chlorite levels, continuous renal replacement therapy was initiated. We prescribed a relatively high dose of CVVH (50 mL/kg) to maximize clearance of chlorite. Pure hemofiltration was chosen over dialysis in an effort to enhance clearance of free hemoglobin and limit renal tubular damage. It is, however, unlikely that removal of free hemoglobin altered the course of AKI as the patient was already anuric.

Although not previously used in chlorite intoxication, red cell exchange has been used in numerous chlorate ingestions, with variable success [6, 8]. We undertook red cell exchange to reduce the burden of red cells affected by chlorite. Indeed, the patient did significantly improve with this intervention. It is unclear if his subsequent deterioration was due to residual abnormal red cells, serum chlorite affecting transfused red cells, or progressive DIC from the persistent presence of hemolytic waste products in the plasma [6]. A second red cell exchange was therefore performed together with a plasma exchange, resulting in cessation of hemolysis and continued physiologic stability.

In conclusion, it is important to consider sodium chlorite ingestion when encountered by a patient with hemolysis, with or without methemoglobinemia. Methylene blue is ineffective for reducing MetHb. Although no clinical evidence exists for its use, high-dose *N*-acetylcysteine may be considered for its theoretical benefit along with minimal risk.

This is the first reported case of sodium chlorite toxicity managed with hemodialysis, hemofiltration, and concurrent red cell and plasma exchange. Without larger case series or prospective studies, it is difficult to suggest this as standard of care; but with physiologic reasoning, our successful patient outcome, and minimal risk, it should be strongly considered in the critically ill patient with related exposure.
